# Discovery of novel [FeFe]-hydrogenases for biocatalytic H_2_-production†
†Electronic supplementary information (ESI) available: Including experimental details and additional electrochemistry and EPR data. See DOI: 10.1039/c9sc03717a


**DOI:** 10.1039/c9sc03717a

**Published:** 2019-09-23

**Authors:** Henrik Land, Pierre Ceccaldi, Lívia S. Mészáros, Marco Lorenzi, Holly J. Redman, Moritz Senger, Sven T. Stripp, Gustav Berggren

**Affiliations:** a Molecular Biomimetics , Department of Chemistry – Ångström Laboratory , Uppsala University , Box 523 , Uppsala , SE-75120 , Sweden . Email: Gustav.Berggren@kemi.uu.se; b Institute of Experimental Physics, Experimental Molecular Biophysics , Freie Universität Berlin , Arnimallee 14 , Berlin , DE-14195 , Germany

## Abstract

A new screening method for [FeFe]-hydrogenases is described, circumventing the need for specialized expression conditions as well as protein purification for initial characterization. [FeFe]-hydrogenases catalyze the formation and oxidation of molecular hydrogen at rates exceeding 10^3^ s^–1^, making them highly promising for biotechnological applications. However, the discovery of novel [FeFe]-hydrogenases is slow due to their oxygen sensitivity and dependency on a structurally unique cofactor, complicating protein expression and purification. Consequently, only a very limited number have been characterized, hampering their implementation. With the purpose of increasing the throughput of [FeFe]-hydrogenase discovery, we have developed a screening method that allows for rapid identification of novel [FeFe]-hydrogenases as well as their characterization with regards to activity (activity assays and protein film electrochemistry) and spectroscopic properties (electron paramagnetic resonance and Fourier transform infrared spectroscopy). The method is based on *in vivo* artificial maturation of [FeFe]-hydrogenases in *Escherichia coli* and all procedures are performed on either whole cells or non-purified cell lysates, thereby circumventing extensive protein purification. The screening was applied on eight putative [FeFe]-hydrogenases originating from different structural sub-classes and resulted in the discovery of two new active [FeFe]-hydrogenases. The [FeFe]-hydrogenase from *Solobacterium moorei* shows high H_2_-gas production activity, while the enzyme from *Thermoanaerobacter mathranii* represents a hitherto uncharacterized [FeFe]-hydrogenase sub-class. This latter enzyme is a putative sensory hydrogenase and our *in vivo* spectroscopy study reveals distinct differences compared to the well established H_2_ producing HydA1 hydrogenase from *Chlamydomonas reinhardtii*.

## Abstract 2

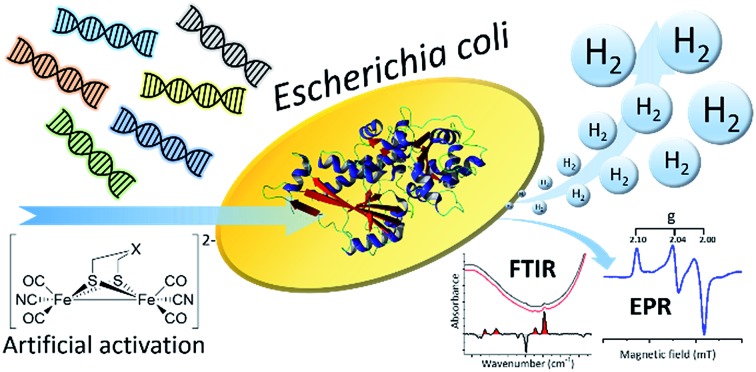
A semi-synthetic screening method for mining the biodiversity of [FeFe]-hydrogenases, expanding the toolbox for biocatalytic H_2_-gas production.

## Introduction

Molecular hydrogen (H_2_) is broadly accepted as one of the most promising energy vectors to replace fossil fuels in a future sustainable society. With its superior gravimetric energy density (approximately three times higher than gasoline)^1^ and clean combustion to H_2_O, it is a good option for storing energy originating from renewable but intermittent sources like solar, wind and wave power. There are however drawbacks hampering the implementation of H_2_ as a general energy carrier, such as the lack of sustainable production methods.^2^ Currently, the industrial standard for producing H_2_ is non-renewable steam methane reforming, which produces CO_2_ as a by-product. New methods for the production of H_2_ are therefore needed, relying on catalysts based on cheap and abundant elements.

Biocatalysis has positioned itself as a major player in sustainable large-scale production of both fine- and bulk chemicals.^3^,^4^ The capacity of enzymes to catalyze chemical transformations with remarkable efficiency, specificity and selectivity make them highly relevant also in an energy context. Moreover, biocatalysts are attractive from a green chemistry point of view due to their ability to perform efficient catalysis at ambient temperatures in aqueous solution, without relying on noble metals. Hydrogenases are enzymes that catalyze the reversible reduction of protons to H_2_.^5^ The most promising hydrogenase for biotechnological application is [FeFe]-hydrogenase due to its remarkable H_2_-production activity with turnover frequencies as high as 9000 s^–1^.^6^ Enzymes from this class of hydrogenases are primarily found in anaerobic bacteria and some green algae. They are dependent on a hexanuclear iron cofactor, commonly referred to as the H-cluster, for catalysis.^5^ The H-cluster consists of a [4Fe-4S]-cluster coupled to a diiron complex, the [2Fe] subsite, *via* a bridging cysteine residue. The low valent metals of the [2Fe] subsite are coordinated by CO and CN^–^ ligands and bridged by an azapropanedithiolate ligand (^–^SCH_2_NHCH_2_S^–^, adt).

The unique nature of the H-cluster in combination with its oxygen sensitivity results in difficulties when expressing [FeFe]-hydrogenases, as common and well-known expression hosts like *Escherichia coli* (*E. coli*) do not natively produce any [FeFe]-hydrogenases and therefore lack the [2Fe] subsite maturation machinery (HydEFG). Thus, standard over-expression techniques result in the synthesis of an inactive apo-enzyme, *i.e.* [FeFe]-hydrogenase harbouring only the active site [4Fe-4S]-cluster but lacking the [2Fe] subsite. To some extent, this challenge can be overcome by utilizing specific *E. coli* strains, co-expressing the [FeFe]-hydrogenase specific maturases needed to synthesize the [2Fe] subsite and deliver it to the active site of the enzyme.^7^,^8^ Alternatively, techniques have now been developed for the preparation of semi-synthetic hydrogenases, circumventing the need for the maturation machinery. The apo-enzyme can be anaerobically purified from *E. coli*, followed by artificial maturation of the apo-hydrogenase with a synthetic mimic of the [2Fe] subsite, [Fe_2_(adt)(CO)_4_(CN)_2_]^2–^ ([2Fe]^adt^), forming a fully active holo-enzyme.^9^–^13^ Still, extensive work is needed to obtain sufficient quantities of purified enzyme to perform artificial maturation and characterization. As a consequence, only a few [FeFe]-hydrogenases are currently characterized,^11^–^18^ despite the diverse nature of this enzyme family.^19^–^23^ All [FeFe]-hydrogenases feature the central H-domain, containing the aforementioned H-cluster. In addition, several sub-classes have been identified on genomic level, ranging from monomeric enzymes with one domain to multimeric enzymes with up to nine distinct domains. The influence of these additional domains on the activity and stability of the enzyme is still largely unknown. In order to establish the viability of [FeFe]-hydrogenase in a biotechnological context, *e.g.* as catalysts for H_2_-production, discovery of novel enzymes needs to become more effective to expand the toolbox of available [FeFe]-hydrogenases.

Recently, we have shown that artificial maturation of the [FeFe]-hydrogenase from *Chlamydomonas reinhardtii* (*Cr*-HydA1) can be performed *in vivo* by supplying [2Fe]^adt^ directly to living cells heterologously expressing the hydrogenase apo-enzyme. This results in *Cr*-HydA1 promoted H_2_-production in both *E. coli* as well as the cyanobacterium *Synechocystis* sp. 6803.^24^,^25^ Moreover, we have reported how the cofactor of the resulting semi-synthetic enzyme can be monitored *in vivo* by electron paramagnetic resonance (EPR).^26^

Herein we present how the combination of artificial maturation and biophysical characterization under *in vivo* conditions can be turned into a tool for efficient screening of novel [FeFe]-hydrogenases. The method is applicable to a range of *E. coli* expression and growth conditions, and allows for basic characterization without the need for time-consuming protein purification. We have also expanded the method by including whole-cell Fourier transform infrared (FTIR) spectroscopy^27^ as well as protein film electrochemistry on non-purified cell lysates. To our knowledge, this is the first time the latter has been reported, and they both provide strong complementary additions to the presented method for discovery and characterization of novel [FeFe]-hydrogenases. More specifically, the screening allowed us to identify a representative enzyme of the hitherto uncharacterized M2e sub-class. This putative sensory hydrogenase was compared to the previously studied [FeFe]-hydrogenase from *Chlamydomonas reinhardtii* as well as a new example from the M2 sub-class.

## Results and discussion

**Fig. 1:**
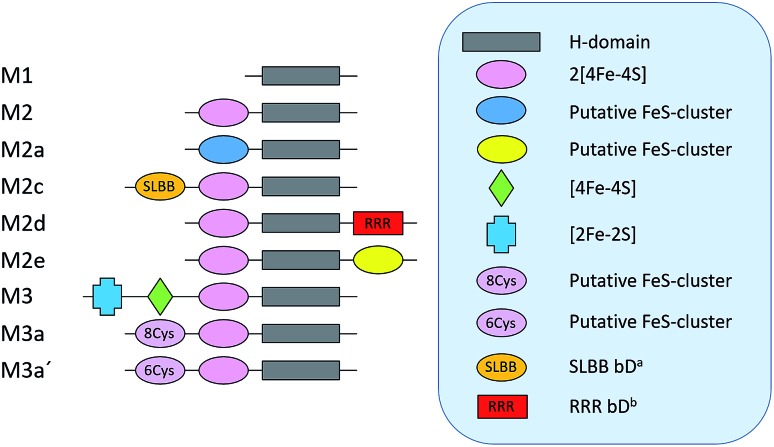
Schematic representation of the various domains present in the eight sub-classes of putative [FeFe]-hydrogenases subject of this study (M2 and M3 enzymes). *C. reinhardtii* HydA1, representing a ninth additional sub-class (M1), was added as a positive control. ^*a*^ Soluble-ligand-binding β-grasp binding domain. ^*b*^ Rubredoxin–rubrerythrin–rubredoxin binding domain. The nomenclature was adapted from Meyer (2007)^20^ and Calusinska *et al.* (2010).^21^

As a proof of concept, we have screened eight hitherto uncharacterized putative [FeFe]-hydrogenases, each originating from a different monomeric sub-class (Fig. 1). These specific sub-classes were chosen for investigation based on earlier bioinformatic investigations (Fig. 1), and the majority are so far completely uncharacterized.^19^–^23^ The well-studied [FeFe]-hydrogenase from *C. reinhardtii* (*Cr*-HydA1) belonging to sub-class M1 (sub-class nomenclature is derived from Meyer^20^ and Calusinska *et al.*^21^) was included as a positive control as it has previously been shown to work under the presented *in vivo* conditions.^24^,^26^ M1 is the structurally simplest known [FeFe]-hydrogenase sub-class consisting only of the H-domain (Fig. 1). Putative [FeFe]-hydrogenase encoding genes from each sub-class were identified by using the protein basic local alignment search tool (pBLAST)^28^ with previously published [FeFe]-hydrogenase sequences as templates,^17^,^20^,^21^,^29^ and one gene from each sub-class was arbitrarily chosen. Amino acid sequences of the putative [FeFe]-hydrogenases were analysed using the Protein Subcellular Localization Prediction Tool (PSORT),^30^,^31^ and all enzymes except for one were predicted to be soluble. The enzyme from sub-class M3a′ was predicted to be membrane bound with a relatively low probability. However, as the predicted transmembrane region includes an iron-sulfur (FeS) binding motif identical to the well-known F-clusters identified in several [FeFe]-hydrogenases, the gene was still included in the screening under the assumption that it is soluble. The genes were synthesized, codon-optimized for expression in *E. coli* and subsequently cloned into a pET-11a(+) vector by Genscript®.

**Fig. 2:**
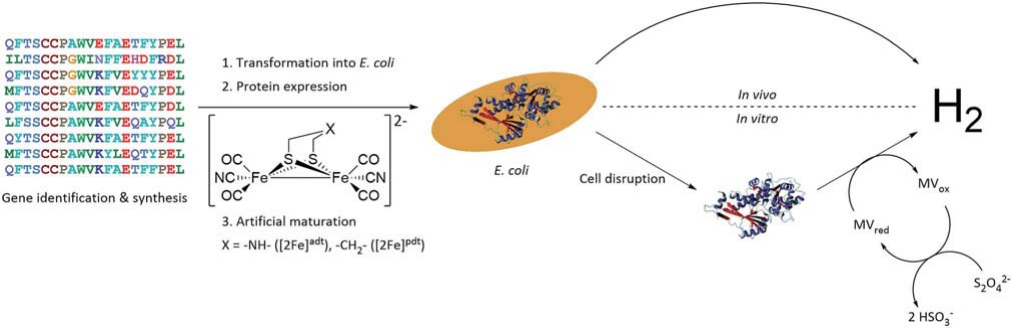
Representation of the workflow from gene identification to H_2_-production, either *via in vivo* or *in vitro* activity assays. *C. reinhardtii* HydA1 is used as a representative 3D protein structure (PDB ID: ; 3LX4).

A small-scale initial screen for H_2_-production was performed by expressing the putative [FeFe]-hydrogenases in 200 mL cultures of *E. coli* cells. Following a standard aerobic over-expression protocol, the apo-hydrogenases were activated *in vivo* with addition of [2Fe]^adt^ to the growth medium under anaerobic conditions. *In vivo* H_2_-production was examined and cells were thereafter subjected to lysis and the cell lysate was investigated for *in vitro* H_2_-production. The *in vitro* assay utilized a previously published protocol using reduced methyl viologen as electron donor (Fig. 2).^32^ The robustness of the artificial maturation method was probed using *Cr*-HydA1 in a range of expression conditions and cell media, and no limitations were found in this initial screening (Table S1†). Still, for the purpose of enzyme screening, each gene was expressed using two different plasmid constructs. They were either cloned in pET-11a(+) with an N-terminal StrepII-tag or in pMAL-c4x with an N-terminal StrepII-tag and a C-terminal maltose binding protein fusion-tag. The latter was added to increase solubility of potentially insoluble proteins. Every construct was expressed in two different *E. coli* strains, a strain optimized for expression of FeS-cluster proteins (BL21(DE3) Δ*iscR*), as well as standard BL21(DE3). Activities in this initial screen are presented in Table 1 as relative activities *versus Cr*-HydA1. The latter hydrogenase had the highest activity under these conditions, while many of the other putative [FeFe]-hydrogenases did not display any significant activity. Albeit these low activity hits are indicative of an active [FeFe] hydrogenase (trace activities indicated as (+) in Table 1), they were close to the H_2_-detection limit of the gas chromatograph and were therefore omitted in the next stage. As all proteins show a high expression, at least when expressed in BL21(DE3) (Fig. S1†), the lack of activity is most likely attributable to low protein solubility (Fig. S2†). Indeed, the majority of the screened enzymes did show at least trace activity when fused with the maltose binding protein. Other factors might include misannotation of genes, incomplete incorporation of FeS-clusters or slow H-cluster formation. These latter factors are however less likely to influence the outcome of the screening as the motifs required for a gene to encode for an [FeFe]-hydrogenase are well defined^19^–^23^ and the *E. coli* BL21(DE3) Δ*iscR* strain has in several cases been shown to successfully incorporate FeS-clusters in multi domain [FeFe]-hydrogenases.^11^,^13^,^33^ Also, slow formation of the H-cluster has so far only been shown in one specific dimeric [FeFe]-hydrogenase from *Desulfovibrio desulfuricans*.^11^ Still, two new active hydrogenases were clearly identified, derived from *Solobacterium moorei* (*Sm*-HydA) and *Thermoanaerobacter mathranii* (*Tam*-HydA), respectively (indicated in bold in Table 1).

**Table 1:** Relative H_2_-production activities of all screened putative [FeFe]-hydrogenases compared to *Cr*-HydA1. Every additional + represents an approximate 10-fold increase in activity. “–” no activity detected; “(+)” trace activity detected. See Table S2 for NCBI accession IDs for all screened [FeFe]-hydrogenases

[FeFe]-Hydrogenase sub-class	pET-11a(+)				pMAL-c4x			
	BL21(DE3)		BL21(DE3) Δ*iscR*		BL21(DE3)		BL21(DE3) Δ*iscR*	
	*In vivo*	*In vitro*	*In vivo*	*In vitro*	*In vivo*	*In vitro*	*In vivo*	*In vitro*
M1 (*Cr*-HydA1)	+++	+++	++	+++	+++	+++	+++	+++
**M2 (*Sm*-HydA)**	**(+)**	**++**	**–**	**++**	**–**	**++**	**–**	**++**
M2a	(+)	**–**	**–**	**–**	**–**	(+)	**–**	(+)
M2c	**–**	**–**	**–**	**–**	**–**	(+)	**–**	**–**
M2d	**–**	**–**	**–**	(+)	(+)	(+)	**–**	**–**
**M2e (*Tam*-HydA)**	**–**	**+**	**–**	**–**	**–**	**(+)**	**–**	**–**
M3	**–**	**–**	**–**	**–**	**–**	**–**	**–**	**–**
M3a	(+)	**–**	**–**	**–**	**–**	**–**	**–**	**–**
M3a′	**–**	**–**	**–**	**–**	**–**	(+)	**–**	**–**

According to the sequence analysis the *Sm*-HydA enzyme belongs to sub-class M2 and it contains an N-terminal domain featuring two [4Fe-4S]-cluster binding motifs, in addition to the H-domain (Fig. 1). *Sm*-HydA is homologous to the previously characterized [FeFe]-hydrogenase from *Megasphaera elsdenii*^12^,^34^ (58% amino acid sequence identity), which also belongs to sub-class M2. *Sm*-HydA shows a 5–10 fold lower activity compared to *Cr*-HydA1 in the *in vitro* H_2_-production assay in all four screened conditions (Table 1).

*Tam*-HydA belongs to sub-class M2e and features the same domains as the aforementioned sub-class M2 hydrogenases. In addition, it also has an uncharacterized C-terminal domain with a conserved four-cysteine motif (Cx_2_Cx_4_Cx_16_C), characteristic of an FeS-cluster binding site. Enzymes belonging to sub-class M2e are putative sensory hydrogenases, previously denoted as HydS.^13^,^35^ On genome level *Tam*-HydA shows some similarity to a recently characterized sensory [FeFe]-hydrogenase from *Thermotoga maritima*.^13^ The latter enzyme has an additional C-terminal PAS (Per-Arnt-Sim) sensory domain commonly involved in signal transduction and belongs to sub-class M2f.^21^ As the PAS domain is lacking in *Tam*-HydA we will retain the HydA classification in the following text, as the sensory function remains to be verified. *Tam*-HydA cloned in pET-11a(+) and expressed in *E. coli* BL21(DE3) shows a 200-fold lower H_2_-production activity *in vitro* compared to *Cr*-HydA1 (Table 1).

*Sm*-HydA and *Tam*-HydA were further investigated with regards to activity and spectroscopic properties. These follow-up studies were performed in *E. coli* BL21(DE3) with the genes cloned in pET-11a(+), as this condition provided activity for both enzymes in the initial screening. Thus, it allowed a comparison of the enzymes under the same conditions, and in the absence of bulky solubility tags.

**Fig. 3:**
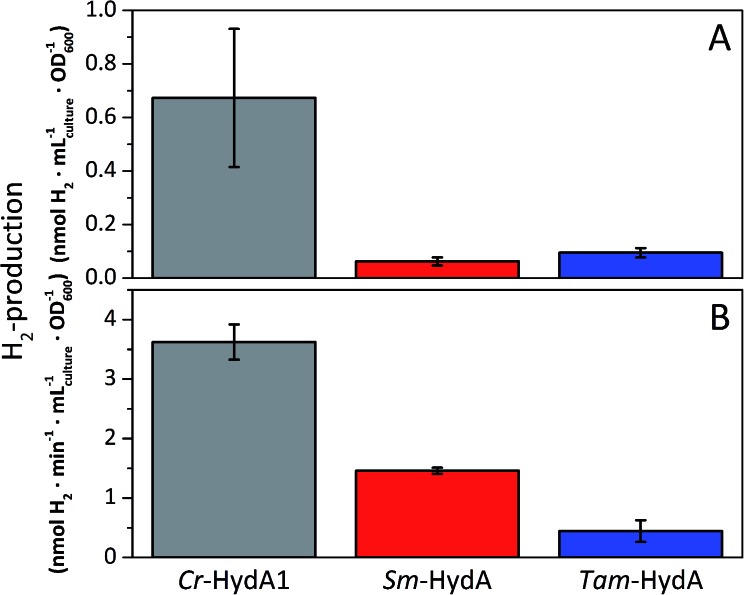
*In vivo* (A) and *in vitro* (B) H_2_-production activities of *Sm*-HydA and *Tam*-HydA compared to the positive control *Cr*-HydA1. *In vivo* H_2_-production was performed in glucose supplemented (0.4%) M9 media. *In vitro* H_2_-production from cell lysates was performed in potassium phosphate buffer (100 mM, pH 6.8, 10 mM methyl viologen, 20 mM sodium dithionite and 1% (v/v) Triton X-100).

A more detailed activity assessment with larger *E. coli* cultures was performed to quantify H_2_-production using the same assays as before (Fig. 2). As shown in Fig. 3A, *in vivo* H_2_-production was clearly observable under these conditions for both *Sm*-HydA and *Tam*-HydA, due to larger culture volumes and higher cell densities. The two enzymes display *in vivo* H_2_-production activities (0.062 ± 0.015, *Sm*-HydA, and 0.095 ± 0.018, *Tam*-HydA, nmol H_2_ per mL_culture_ per OD_600_) that are about eleven and seven times lower than *Cr*-HydA1 (0.67 ± 0.26 nmol H_2_ per mL_culture_ per OD_600_), respectively. Conversely, the *in vitro* H_2_-production activity shows a different pattern (Fig. 3B). *Cr*-HydA1 is still the best H_2_-producer at 3.6 ± 0.30 nmol H_2_ per min per mL _culture_ per OD_600_ and similarly to the *in vivo* assays *Tam*-HydA has an eight times lower activity at 0.45 ± 0.18 nmol H_2_ per min per mL_culture_ per OD_600_. However, *Sm*-HydA has an activity of 1.5 ± 0.052 nmol H_2_ per min per mL_culture_ per OD_600_, *i.e.* approximately 40% of the activity of *Cr*-HydA1. It remains unclear as to why the activity of *Sm*-HydA increases relative to the other enzymes following cell lysis. Sodium dithionite was added during *in vivo* activation in an attempt to simulate the reductive conditions of the *in vitro* assay but it showed no effect on the relative activities. This behaviour is therefore likely reflecting differences between the [FeFe]-hydrogenases in their affinity for the available electron donors in *E. coli* or the artificial electron donor methyl viologen.

**Fig. 4:**
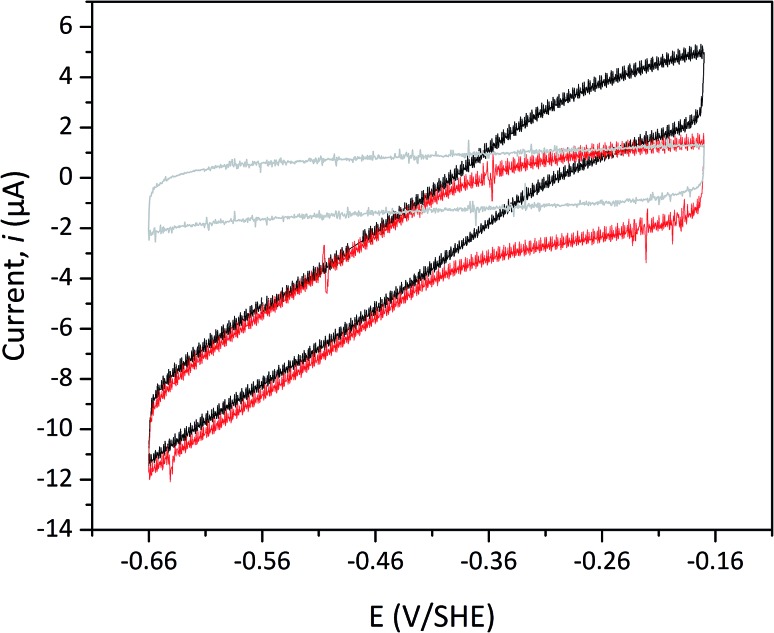
Cyclic voltammogram of *Sm*-HydA containing cell lysate from *E coli*. Analysis was performed either under H_2_ (black) or Ar (red) at pH 6.0 and room temperature. The cyclic voltammogram recorded for *Tam*-HydA under H_2_ shown in grey. Cycles start at –0.42 V *vs.* SHE. Potential step 0.5 mV, scan rate 5 mV s^–1^, electrode rotation speed 3k rpm.

**Fig. 5:**
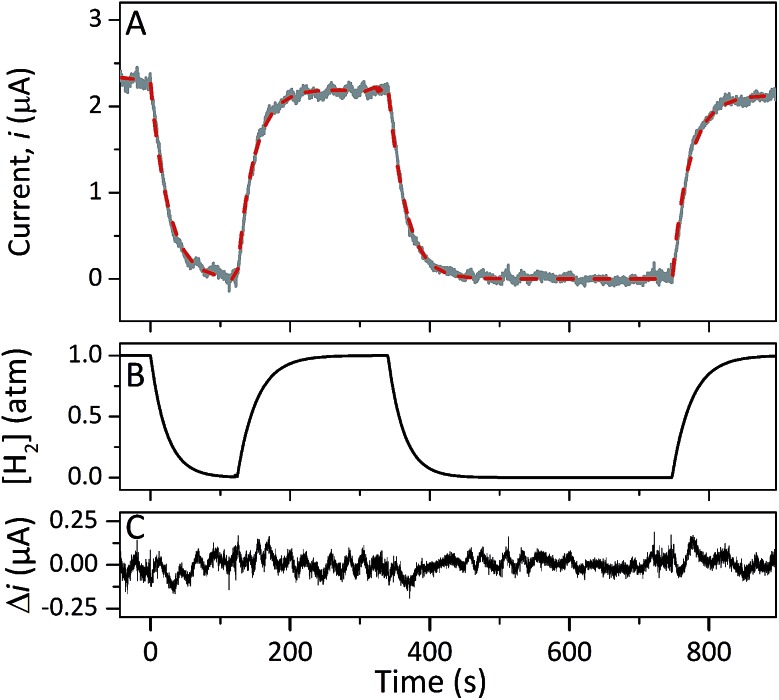
Chronoamperometry of *Sm*-HydA containing cell lysate from *E. coli* at a potential of –0.2 V/SHE. Current was measured over-time as a function of H_2_-pressure (A). H_2_-pressure was repeatedly varied between 0–1 atm (B), leading to a variation in H_2_-oxidation activity (A, grey trace). A Michaelis–Menten fit was performed (A, red dashed line) and returned a *K*_M_ = 4; given the experimental concentration range we conclude that *K*_M_ (H_2_) is >1 atm H_2_. The difference between the data and the fit was calculated (C). Solution at pH 7.0 and 25 °C, electrode rotation speed 3k rpm.

Protein film electrochemistry was applied in order to gain further insight into the reactivity of the enzymes. The analysis was performed on non-purified cell lysates, following spontaneous adsorption of the enzymes onto carbon nanotube coated electrodes. No hydrogenase activity was detected for *Tam*-HydA under these conditions (Fig. 4, grey trace), either due to insufficient binding to the electrode surface or low activity of *Tam*-HydA under these conditions. However, the activity of *Cr*-HydA1 and *Sm*-HydA was readily detected and could be analysed and compared. Cyclic voltammetry traces of the two latter enzymes display clear catalytic waves corresponding to H_2_-production and oxidation (Fig. 4 and S3†). Currents indicative of H_2_-production were detected both under 1 atm H_2_ and 1 atm Ar, while the catalytic wave attributable to H_2_-oxidation is clearly absent under Ar. A sustained current was observed in chronoamperometry experiments performed under a H_2_ atmosphere at an oxidizing potential, attributable to the oxidation of H_2_, and the *Sm*-HydA enzyme was stable on the electrode surface on the time-scale of the experiment (minutes) (Fig. 5A, grey trace). H_2_ partial pressure was varied between 1 and 0 atm by switching between H_2_- and Ar-bubbling (Fig. 5B). As a result, the activity decreased and increased following the relative substrate availability (Fig. 5A, grey trace). This trace was modelled with the Michaelis–Menten equation, where the substrate concentration is time-dependent (Fig. 5A, red dashed line).^36^,^37^ Here, *K*_M_ could only be determined as >1 atm H_2_, as the experimental setup did not allow use of pressures >1 atm. This shows that *Sm*-HydA has a lower affinity for H_2_ than *Cr*-HydA1, for which a *K*_M_ of 0.57 ± 0.15 atm H_2_ was determined (Fig. S4†), in agreement with the previously published value for the purified enzyme of 0.64 ± 0.05 atm H_2_.^36^ The higher *K*_M_ for *Sm*-HydA suggests an improved bias towards H_2_-production over H_2_-oxidation, as compared to *Cr*-HydA1.

**Fig. 6:**
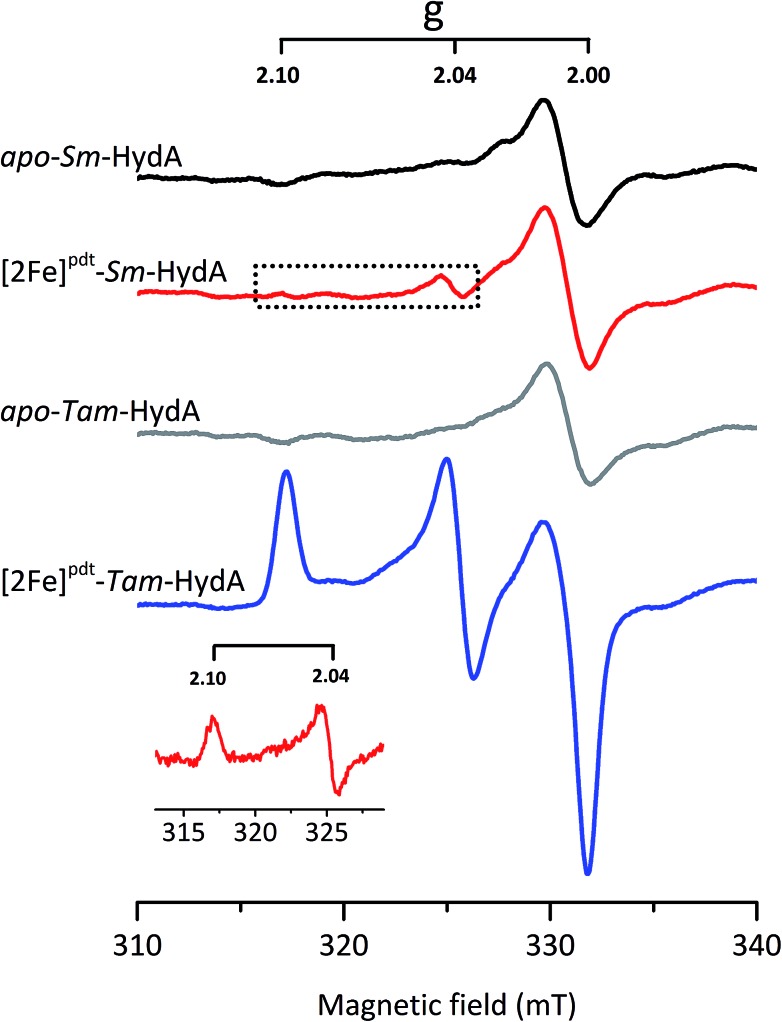
*In vivo* H-cluster assembly in *E. coli* monitored using X-band EPR spectroscopy. A rhombic EPR signal characteristic of the H_ox_ state is clearly observable in [2Fe]^pdt^-*Tam*-HydA (*g* = 2.10; 2.04; 2.00); two of these peaks are also apparent in [2Fe]^pdt^-*Sm*-HydA following subtraction of the cell background signals. Samples were collected from cells incubated in the absence (apo-*Sm*-HydA and apo-*Tam*-HydA) and presence of [2Fe]^pdt^. Inset: [2Fe]^pdt^-*Sm*-HydA spectrum corrected for contribution from the cells by subtracting the apo-*Sm*-HydA signal. EPR experimental conditions: *T* = 10 K, *P* = 1 mW, *ν* = 9.28 GHz.

EPR spectroscopy is a sensitive spectroscopic technique for studying [FeFe]-hydrogenase, due to the characteristic signals of the H-cluster.^5^,^26^ Thus we explored the possibility to utilize whole-cell X-band EPR spectroscopy in the presented screening to directly verify the presence of the enzyme. In order to facilitate the detection of the H-cluster, this study was performed using [2Fe]^adt^ as well as an alternative [2Fe] subsite mimic, [2Fe]^pdt^ (pdt = propanedithiolate). The [2Fe]^pdt^ cofactor mimic lacks the nitrogen bridgehead, resulting in a loss of catalytic rate and accumulation of an oxidized paramagnetic state (H_ox_).^9^,^26^,^38^ EPR spectra recorded of whole-cell samples containing only the overproduced apo-hydrogenases (Fig. 6, S5 and S6,† apo-*Sm*-HydA and apo-*Tam*-HydA) did not reveal any enzyme specific EPR signal(s). Similarly, apo-hydrogenase containing cells incubated with the [2Fe]^adt^ complex did not reveal any well-defined new signal in the case of *Sm*-HydA, while maturation of *Tam*-HydA with [2Fe]^adt^ resulted in a complex signal containing a mixture of different EPR active species (Fig. S5†). Contributions from an H_ox_-like state to the *Tam*-HydA spectrum is visible on the *g* = 2.10 feature, and additional signals at *g* = 2.03 and a broad *g* ≈ 1.90 feature show similarities to signals previously observed for the *Thermotoga maritima* sensory [FeFe]-hydrogenase.^13^ Still, well-defined H-cluster signals were not readily apparent in either of the [2Fe]^adt^ treated samples. Conversely, distinct H-cluster signals for both *Sm*-HydA and *Tam*-HydA could be detected in cells after incubation with the [2Fe]^pdt^ cofactor. Whole cell samples of [2Fe]^pdt^-*Tam*-HydA display a well-defined rhombic signal (*g*_*zyx*_ = 2.10, 2.04, 2.00) comparable to previously published data on identically treated *Cr*-HydA1 (Fig. 6, blue spectrum and Fig. S6†). It is therefore assigned to an H_ox_-like state.^26^ The signals for *Sm*-HydA were weak, preventing the identification of all *g*-values. Still, features at *g* = 2.10 and 2.04, attributable to an H_ox_-like state were discernable also in [2Fe]^pdt^-*Sm*-HydA containing cells (Fig. 6, red spectrum and inset, and Fig. S6†). Considering the intense EPR signal observed for [2Fe]^pdt^-*Tam*-HydA and the high expression level of apo-*Sm*-HydA (Fig. S1†), the weak EPR-signal observed for the latter enzyme is most likely due to low solubility of the overproduced protein (Fig. S2†), ineffective FeS-cluster incorporation or incomplete H-cluster assembly. Alternatively, it could be due to a thermodynamic preference towards an EPR silent state. Nevertheless, EPR spectroscopy of [2Fe]^pdt^ treated cells verified the successful assembly of a semi-synthetic H-cluster in both enzymes.

**Fig. 7:**
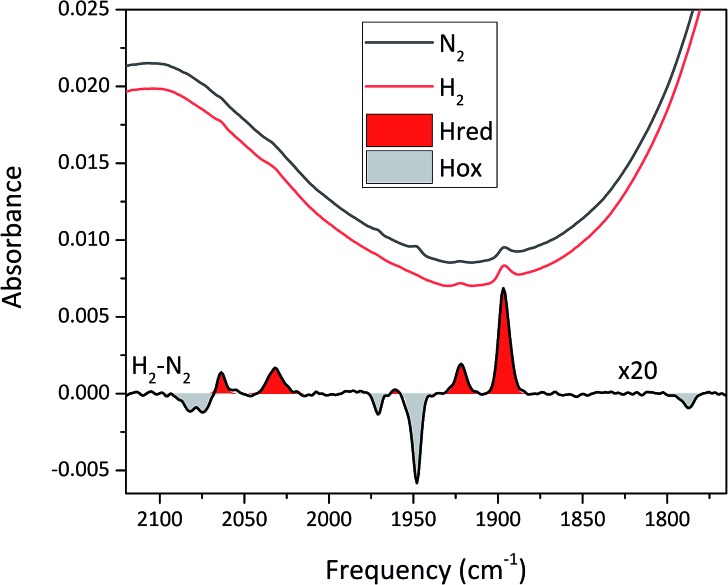
*In situ* ATR FTIR spectra of *Tam*-HydA containing *E. coli* cells activated with [2Fe]^adt^. Absolute spectra under N_2_ (black) and H_2_ (red) are shown above a H_2_–N_2_ difference spectrum (magnified 20×). Note the presence of H_red_ (*e.g.* the reporter peak at 1896 cm^–1^), even under N_2_.

To circumvent the limitations of EPR spectroscopy in detection of all catalytic states, we also employed whole-cell FTIR spectroscopy. The absorption bands of the H-cluster CN^–^ and CO ligands are typically exploited to track changes in cofactor geometry as well as in redox- and protonation states.^5^ For *Sm*-HydA, no cofactor ligand band signal could be detected, further indicating its low concentration in the *E. coli* cells. Fig. 7 reports on the absorption spectrum of *E. coli* cells containing *Tam*-HydA activated with [2Fe]^adt^ recorded by *in situ* attenuated total reflectance (ATR) FTIR spectroscopy in the CN^–^ and CO ligand frequency regime. The absolute spectra were recorded at pH 8 under N_2_- and H_2_-atmosphere and the main CO bands of the cofactor were clearly detectable. As prepared, the enzyme adopted a redox state with low frequency CO bands that was converted into a species with up-shifted CO bands upon extensive purging with N_2_. In the presence of H_2_, the original signature was immediately restored. The corresponding H_2_–N_2_ difference spectrum (magnified 20-fold) allowed separation of two redox states associated with the different gas atmospheres (band positions in Table 2). In accordance with earlier studies on various [FeFe]-hydrogenases, we assign positive bands to the reduced state H_red_ (red area) and negative bands to H_ox_ (grey area).^13^,^39^–^41^ As can be seen in Fig. 7, the two CN^–^ bands attributed to H_ox_ (2082 and 2074 cm^–1^) are partially overlapping. Also, one of the CO bands assigned to H_red_ (1961 cm^–1^) is barely visible due to the close proximity of nearby CO bands belonging to H_ox_ (1971 and 1948 cm^–1^). The observed formation of H_red_ in the presence of H_2_ provides spectroscopic support for the capacity of *Tam*-HydA to perform H_2_ oxidation. In addition, the slow and incomplete formation of H_ox_ under N_2_ suggests inferior H_2_ release activity. This is accompanied with an unusual persistence of H_red_ that was not observed with *E. coli* cells containing *Cr*-HydA1 (Fig. S7†), suggestive of distinct differences in the reactivity of the enzymes under *in vivo* conditions. Finally, our data also verifies that the whole-cell screening method is compatible with ATR-FTIR spectroscopy, providing a strong complement to the EPR spectroscopy.

**Table 2:** Band positions of FTIR spectra in Fig. 7

Redox state	Cofactor ligand				
	CN^–^	CN^–^	CO	CO	CO
H_ox_ (cm^–1^)	2082	2074	1971	1948	1788^*a*^
H_red_ (cm^–1^)	2063	2032	1961	1921	1896

^*a*^Fe–Fe bridging carbonyl ligand (μCO).

## Conclusions

Herein we present a straightforward method for rapid screening and basic characterization of novel [FeFe]-hydrogenases, compatible with a range of *E. coli* expression conditions. The method is based on *in vivo* artificial maturation of overproduced apo-[FeFe]-hydrogenase with synthetic cofactors and verification of hydrogenase activity through standard *in vitro* and/or *in vivo* activity assays. As presented herein, these enzymatic assays can also be supported by protein film electrochemistry while still avoiding any protein purification. We have also shown that whole-cell EPR and FTIR spectroscopy can be readily employed to complement the activity measurements and verify the successful expression also of apparent low activity [FeFe]-hydrogenases, as exemplified by *Tam*-HydA. Despite low temperature induction and the use of solubility fusion protein constructs, the solubility of the proteins remains a significant challenge. This underscores the need to screen several enzymes to obtain hits suitable for purification and more detailed studies. Still, one of the main advantages of the presented method is that protein expression can be performed without specialized cells or conditions. Additionally, all analysis is carried out on whole cells or non-purified cell lysates, eliminating the need for extensive protein purification. This first proof of concept screening included putative [FeFe]-hydrogenase genes from eight different structural sub-classes, and resulted in the discovery of two previously uncharacterized [FeFe]-hydrogenases. On a methodology level, the activation of M2 (*Sm*-HydA) and M2e (*Tam*-HydA) enzymes under these assay conditions underscore that the method is capable of detecting also complex multi-domain hydrogenases with several FeS-clusters. Thus, the presented method can be expected to facilitate the discovery of novel [FeFe]-hydrogenases, paving the way for understanding their complex chemistry and increasing the toolbox of available biocatalysts applicable in a future H_2_-society. Both *Sm*-HydA and *Tam*-HydA show distinctively different features as compared to *Cr*-HydA1. *Sm*-HydA displays high activity based on activity assays and protein film electrochemistry while spectroscopic data indicates a low concentration of the enzyme. In combination, these results suggest that *Sm*-HydA has a high specific activity, warranting further investigation. *Tam*-HydA, on the other hand, is readily detectable by EPR and FTIR spectroscopy, underscoring that the enzyme expresses well and is readily matured under these conditions. Despite the high intracellular concentration, the enzyme displayed very low H_2_-evolution activities. Moreover, the whole-cell FTIR spectroscopy study of *Tam*-HydA revealed an unexpected stability of H_red_ over H_ox_. These observations support the notion that the latter enzyme indeed serves a sensory rather than catalytic function, as previously proposed for enzymes from sub-class M2e. As *Tam*-HydA is the first reported example of this sub-class, it provides an entry point into studying the reactivity and biological function of this hitherto unstudied type of [FeFe]-hydrogenase.

## Conflicts of interest

There are no conflicts to declare.

## Supplementary Material

Supplementary information
