# Supplementary material for: Discovery of novel [FeFe]-hydrogenases for biocatalytic H2-production
Source: Chem Sci. 2019 Sep 23;10(43):9941–8. doi: 10.1039/c9sc03717a (PMC6984386; doi:10.1039/c9sc03717a)
Supplement: Supplementary file 1 [file SC-010-C9SC03717A-s001.pdf]

Electronic Supplementary Material (ESI) for Chemical Science.
This journal is © The Royal Society of Chemistry 2019

Supplementary Information

Discovery of Novel [FeFe]-Hydrogenases for Biocatalytic H_2-Production

Henrik Land,[a] Pierre Ceccaldi,[a] Lívia S. Mészáros,[a] Marco Lorenzi,[a] Holly J. Redman,[a] Moritz Senger,[b] Sven Stripp [b] and Gustav Berggren *[a]

[a] Molecular Biomimetics, Department of Chemistry – Ångström Laboratory, Uppsala University, Box 523, SE-75120, Uppsala, Sweden

[b] Institute of Experimental Physics, Experimental Molecular Biophysics, Freie Universität Berlin, Arnimallee 14, DE-14195, Berlin, Germany

E-mail: Gustav.Berggren@kemi.uu.se

Table of Contents

Experimental procedures 1
- General 1
- Plasmids 1
- Protein expression and *in vivo* artificial activation 1
- SDS-PAGE and Western blot analysis 1
- H_2-production assays 2
  - *In vivo* 2
  - *In vitro* 2
- Protein film electrochemistry on cell lysates 2
- EPR and FTIR sample preparation 3
- Whole-cell EPR 3
- Whole-cell FTIR 3

Supplementary tables and figures 4
- Table S1. *In vivo* condition screening 4
- Table S2. Screened putative [FeFe]-hydrogenases 5
- Figure S1. SDS-PAGE of overproduced [FeFe]-hydrogenases 6
- Figure S2. Western blot of overproduced [FeFe]-hydrogenases, soluble fraction 7
- Figure S3. Cyclic voltammograms, *Cr*-HydA1 8
- Figure S4. Chronoamperometry, *Cr*-HydA1 H_2-dependence 9
- Figure S5. Whole-cell EPR with $[2Fe]^{adt}$ treated *Sm*-HydA and *Tam*-HydA 10
- Figure S6. Whole-cell EPR with $[2Fe]^{pdt}$ treated *Sm*-HydA and *Tam*-HydA 11
- Figure S7. Whole-cell FTIR spectra 12

Additional information 13
- *Sm*-HydA amino acid sequence 13
- *Tam*-HydA amino acid sequence 13

References 14

Experimental procedures

General

All chemicals were purchased from Sigma-Aldrich or VWR and used as received unless otherwise stated. All anaerobic work was performed in an MBRAUN glovebox ([O_2] < 10 ppm). The $[2Fe]^{adt}$ and $[2Fe]^{pdt}$ subsite mimics were synthesized in accordance to literature protocols with minor modifications, and verified by FTIR spectroscopy.[1-5]

Plasmids

[FeFe]-hydrogenase constructs in pET-11a(+) were synthesised and cloned by Genscript® using restriction sites NdeI and BamHI. The [FeFe]-hydrogenase encoding genes were subsequently cloned into pMAL-c4x using restriction sites EcoRI and BamHI (*Tam*-HydA, M3a and M3a´) or BamHI and HindIII (*Sm*-HydA, M2a, M2c, M2d and M3). The *Cr*-HydA1 gene was expressed in a pET-DUET expression vector.

Protein expression and *in vivo* artificial activation

E. coli containing genes for [FeFe]-hydrogenase expression were grown overnight in LB medium at 37°C. These cultures were subsequently used to inoculate 200 mL of M9 medium (22 mM Na_2HPO_4, 22 mM KH_2PO_4, 85 mM NaCl, 18 mM NH_4Cl, 0.2 mM $MgSO_4$, 0.1 mM $CaCl_2$, 0.4% (v/v) glucose) containing 100 µg/mL ampicillin. Cultures were grown at 37°C and 150 rpm until an optical density (OD_{600}) of appr. 0.4 was reached. Protein expression was induced by addition of 1 mM isopropyl β-D-1-thiogalactopyranoside (IPTG), 0.1 mM $FeSO_4$ was also added at the time of induction, and cultures were incubated at 20°C and 150 rpm for appr. 16 h. Cells were thereafter harvested by centrifugation in a Beckman Coulter Avanti J-25 centrifuge (5000 rpm/4424 xg, 10 min). Cells were resuspended in 1 mL M9 medium and sparged with N_2 before being transferred to the glovebox. 0.5 mL of the resuspended cells were then diluted with 1.5 mL of additional M9 medium. Cofactor incorporation was then performed by addition of 100 µg $[2Fe]^{adt}$ or $[2Fe]^{pdt}$ subsite mimic (final concentration 80 µM).

SDS-PAGE and Western blot analysis

Protein expression was analyzed by 12% SDS-PAGE minigels in a Mini-PROTEAN® Tetra System (Bio-Rad) system. The proteins were stained with Page Blue protein staining solution (Thermo Fisher Scientific) according to the supplier instructions. For Western blot, proteins were blotted onto a PVDF membrane using a Trans-Blot® Turbo™ Transfer Pack (Bio-Rad) at 1.0 A for 30 min in a Trans-Blot® Turbo™ Transfer System (Bio-Rad). For StrepII-tagged proteins (M2-M3a´), the blotted membrane was treated overnight with Strep-Tactin® HRP conjugate (IBA) at a 1:100000 dilution. For His-tagged proteins (M1), the blotted membrane was treated with THE™ His Tag Antibody, mAb, Mouse (GenScript) for 1 hour according to supplier instructions and overnight with rabbit-anti-mouse IgG HRP

conjugate (Agrisera) at a 1:5000 dilution. Interactions were detected with Clarity™ Western ECL Substrate (Bio-Rad). Chemiluminescence was measured in a ChemiDoc XRS system (Bio-Rad) using an exposure time of 30 s.

H_2-production assays

In vivo

E. coli cells treated with $[2Fe]^{adt}$ were transferred to an airtight vial and incubated at 37°C and 150 rpm for 1 hr. H_2 production was then determined by analyzing the reaction headspace using a PerkinElmer Clarus 500 gas chromatograph (GC) equipped with a thermal conductivity detector (TCD) and a stainless-steel column packed with Molecular Sieve (60/80 mesh). The operational temperatures of the injection port, the oven and the detector were 100 °C, 80 °C and 100 °C, respectively. Argon was used as carrier gas at a flow rate of 35 mL min^{-1}.

In vitro

Following *in vivo* activation with $[2Fe]^{adt}$, cells were transferred to the glovebox and harvested by centrifugation (13.000 rpm, 4 min). The cells were then washed two times with 1 mL Tris-HCl buffer (100 mM Tris, 150 mM NaCl, pH 7.5) and resuspended in 0.5 mL lysis buffer (30 mM Tris-HCl, 0.2 % (v/v) Triton X-100, 0.6 mg mL^{-1} lysozyme, 0.1 mg mL^{-1} DNAse, 0.1 mg mL^{-1} RNAse). Cell lysis was performed by three cycles of freezing/thawing in liquid N_2 and the supernatant was recovered by centrifugation (13.000 rpm, 10 min). 0.38 mL supernatant was then diluted to 2 mL in potassium phosphate buffer (100 mM, pH 6.8) containing 10 mM methyl viologen, 20 mM sodium dithionite and 1 % (v/v) Triton X-100. Reactions were incubated in 37°C for 15 min. H_2 production was then determined by analyzing the reaction headspace on GC (see above).

Protein film electrochemistry on cell lysates

Protein film electrochemistry experiments were run in an anaerobic glovebox, using a saturated calomel reference electrode (Fisher Scientific) and a 0.5 mm platinum wire (Sigma Aldrich) as counter. Buffer composition was a mixture of MES, CHES, HEPES, TAPS and sodium acetate, 5 mM each, with NaCl (0.1 M) as carrying electrolyte. pH was 6 or 7, as indicated in the figure captions. The working electrodes used for immobilising the lysates were commercial glassy carbon (GC) rotating disk electrodes (Pine research), functionalised with 10-100 nm multi-wall carbon nanotubes (MWCNTS). The functionalisation procedure consisted of preparing a solution of 1-3 mg/mL of MWCNTS in 1,2-dinitropyrolidone followed by homogenisation by sonication for 15 min. Then, the GC surface (r= 1 or 2 mm) was dropcast with 3-5 µL of the MWCNTS and dried for at least 24 h. The GC/MWCNTS electrodes were then transferred to the glovebox and painted with 5-20 µL of cell lysate (prepared in the same way as for the *in vitro* H_2-production assays), dried for up to 20 min, in order to concentrate

the enzyme onto the electrode. Electrochemical data was acquired using an Eco/Chemie PGSTAT10 and the GPES software (Metrohm/Autolab). Data were analysed using Qsoas (qsoas.org).[6]

EPR and FTIR sample preparation

The 2 mL dense cell suspension generated via the *in vivo* artificial activation protocol followed by 1 hr incubation at 37°C were centrifuged and the cell pellet was washed with 1 mL Tris-HCl buffer (100 mM Tris, 150 mM NaCl, pH 7.5) three times under anaerobic conditions. For EPR samples the cells were resuspended with 400 µL TRIS-HCl buffer after the washing protocol and then transferred into EPR tubes. The tubes were capped and directly frozen in liquid N_2. In case of the FTIR samples, four separate 2 mL sample preparations were combined, concentrated and resuspended in 400 µL Tris-HCl buffer and frozen in liquid N_2 under anaerobic conditions.

Whole-cell EPR

Measurements were performed on a Bruker ELEXYS E500 spectrometer using an ER049X SuperX microwave bridge in a Bruker SHQ0601 cavity equipped with an Oxford Instruments continuous flow cryostat and using an ITC 503 temperature controller (Oxford Instruments). Measurement temperature was 10 K, using liquid helium as coolant, with the following EPR settings unless otherwise stated: microwave power 1 mW modulation amplitude 1 mT, modulation frequency 100 kHz. The spectrometer was controlled by the Xepr software package (Bruker).

Whole-cell FTIR

For *in situ* ATR FTIR spectroscopy, 1 µL cell suspension was deposited on the silicon crystal of an ATR cell in the beam path of a commercial FTIR spectrometer (Bruker). All experiments were performed at ambient temperature (~24 °C) and pressure (~1 atm), in the dark, and on hydrated films of physiological pH values (pH ~8). The cell suspension was dried under 100% N_2 gas and re-hydrated with *A. bidest* in the humidified gas stream (aerosol), similar to what was reported for purified protein earlier.[7] Reduction of [FeFe]-hydrogenase in the cells was induced by adding 1% H_2 to the N_2 gas stream (1.5 L min^{-1}). In the absence of H_2, H_{ox} recovered due to auto-oxidation. This latter process was rapid in the case of *Cr*-HydA1, while extended incubation under N_2 was required in the case of *Tam*-HydA. Transitions were followed with a spectral precision of 2 cm^{-1} and 1,000 averages of interferometer scans per spectrum. Difference spectra were calculated by subtraction of a N_2 spectrum from an H_2 spectrum. In the CO/CN^- regime of the H-cluster, negative bands are assigned to Hox whereas positive bands represent Hred.[8]

Supplementary tables and figures

Table S1. *In vivo* condition screening

The robustness of the screening protocol was probed using *Cr*-HydA1, and the results are summarized in Table S1. Two different plasmid constructs were tested, using a standard T7 promoter or a low expression trc promoter. The two constructs were expressed in three different media, LB, TB or M9, to probe the importance of the growth medium. In all cases successful enzyme activation was observed, as determined by the detection of H_2-production.

Cells containing a *Cr*-HydA1 expressing plasmid were grown in 100 mL volume in different media. In one set of the experiments 0.5 mM IPTG was added at the time of the inoculation, and the cells were grown at 37˚C until OD_{600} = 0.2. In the other set of experiments the cells were pre-grown until OD_{600} = 0.2, before the protein overproduction was induced with 0.5 mM IPTG and incubated for 2 hours at 37˚C. In both cases, the cells were then concentrated to 2 mL with addition of fresh, 0.4% glucose supplemented media, and an *in vivo* enzyme activation was performed with addition of 100 µg $[2Fe]^{adt}$ (final concentration 80 µM) under anaerobic conditions. H_2-production was measured after 1 h incubation at 37˚C by analyzing the reaction headspace on GC (see above).

Table S1. Screening of different plasmid constructs and growth conditions for *in vivo* H_2-production.

***in vivo* H_2-producing activity (nmol H_2/OD_{600}/mL)**								
induction at the time of the inoculation						***induction at OD_{600} = 0.2***		
trc promoter[a]			T7 promoter[b]			T7 promoter[a]		
LB[c]	TB[d]	M9[e]	LB[c]	TB[d]	M9[e]	LB[c]	TB[d]	M9[e]
8.55±1.7	17.94±5.5	9.95±0.3	3.34±0.4	7.04±0.6	3.11±0.3	18.74±4.3	25.63±2.7	20.42±4.0

(a) A pET-DUET plasmid was used in this experiment.
(b) A pPMQAK1 plasmid was used in this experiment.
(c) 10g/L bacto-tryptone, 5 g/L yeast extract, 10 g/L NaCl
(d) 20 g/L bacto-tryptone, 24 g/L yeast extract, 4 mL/L glycerol, 17 mM KH_2PO_4, 72 mM K_2HPO_4
(e) 22 mM Na_2HPO_4, 22 mM KH_2PO_4, 85 mM NaCl, 18 mM NH_4Cl, 0.2 mM $MgSO_4$, 0.1 mM $CaCl_2$, 0.4% (v/v) glucose

Table S2. Screened putative [FeFe]-hydrogenases

Table S2. The putative [FeFe]-hydrogenases applied in the screening with their corresponding NBCI accession IDs

Sub-class	NCBI accession ID[a]
M2 (*Sm*-HydA)	WP_040627710
M2a	WP_036203142
M2c	WP_005958327
M2d	WP_023050767
M2e (*Tam*-HydA)	WP_013150113
M3	WP_040952086
M3a	WP_084730690
M3a´	WP_087990317

(a) Accession ID used in the NCBI GenBank.

Figure S1. SDS-PAGE of overproduced [FeFe]-hydrogenases

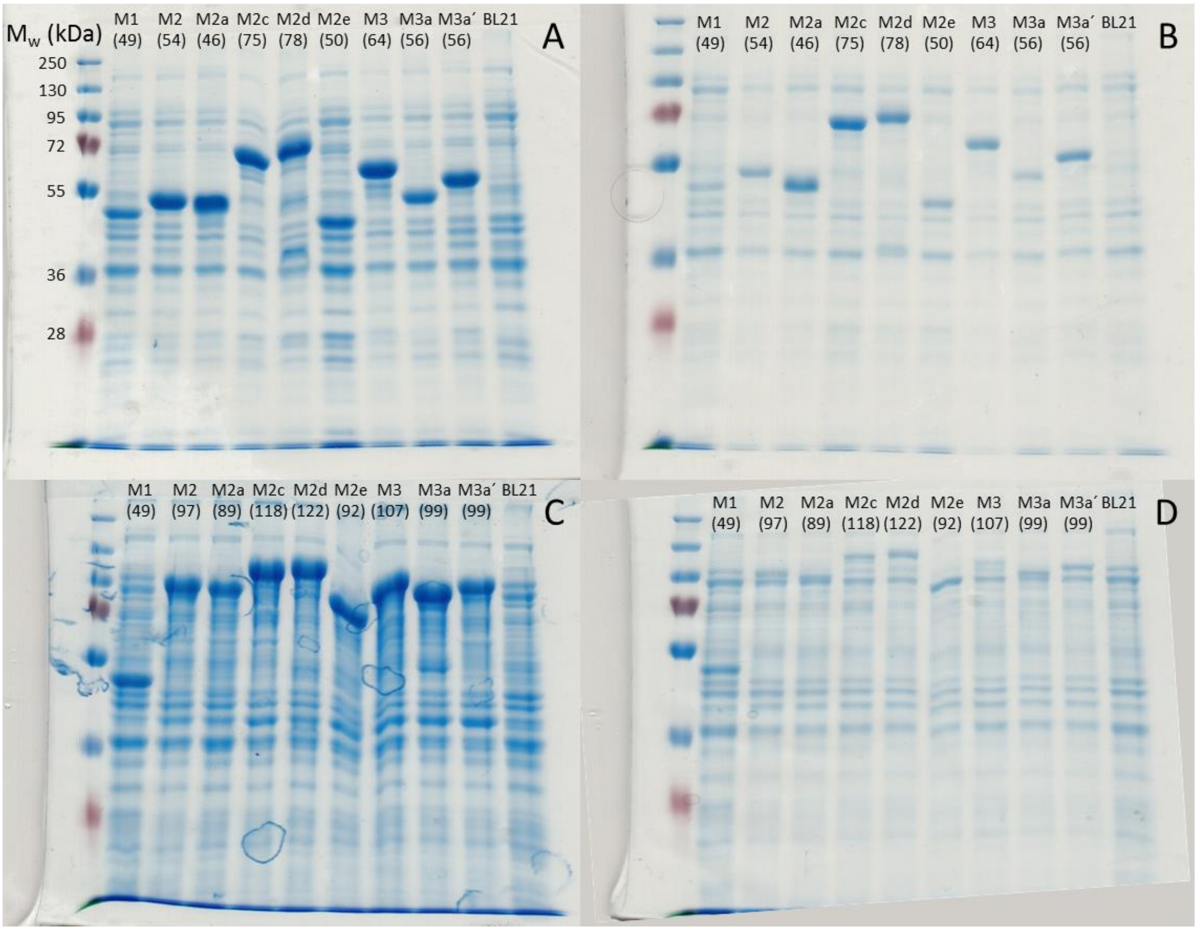

Figure S1. SDS-PAGE of overproduced [FeFe]-hydrogenases from the initial *in vivo*/*in vitro* screening. Expected molecular weights in kDa are indicated in brackets. Sampling was performed before harvesting. The enzymes (M2-M3a´) were expressed and cloned in (**A**) *E. coli* BL21(DE3) and pET-11a(+), (**B**) *E. coli* BL21(DE3) ΔiscR and pET-11a(+), (**C**) *E. coli* BL21(DE3) and pMAL-c4x, (**D**) *E. coli* BL21(DE3) ΔiscR and pMAL-c4x. M1 (*Cr*-HydA1) was cloned in pET-DUET in all cases.

Figure S2. Western blot of overproduced [FeFe]-hydrogenases, soluble fraction

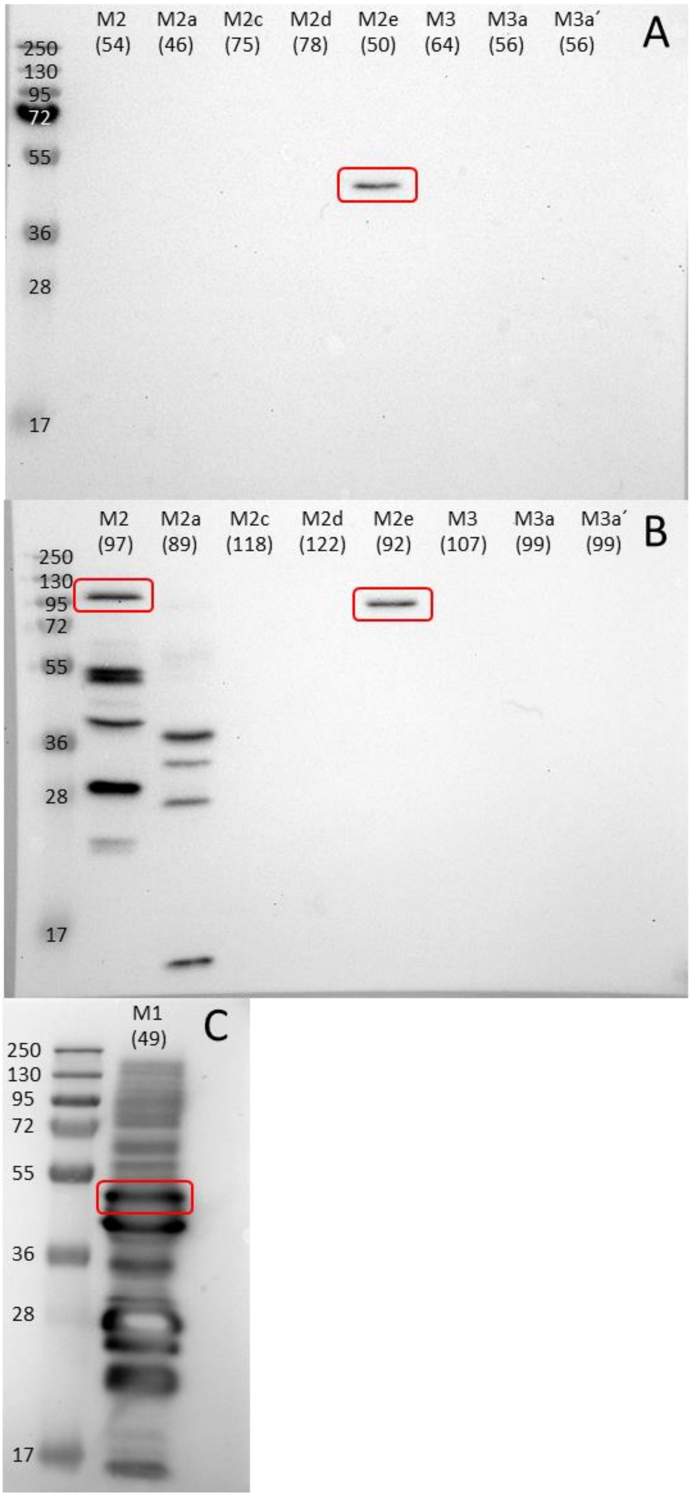

Figure S2. Western blot of overproduced [FeFe]-hydrogenases from the initial in vivo/in vitro screening. Expected molecular weights in kDa are indicated in brackets. Sampling was performed after cell lysis. The enzymes (M2-M3a´) were expressed and cloned in (**A**) E. coli BL21(DE3) and pET-11a(+), (**B**) E. coli BL21(DE3) and pMAL-c4x, (**C**) E. coli BL21(DE3) and pET-DUET. Soluble [FeFe]-hydrogenases are highlighted in red. The M1, M2 and M2e enzymes were observed in the soluble fraction.

Figure S3. Cyclic voltammograms, *Cr*-HydA1

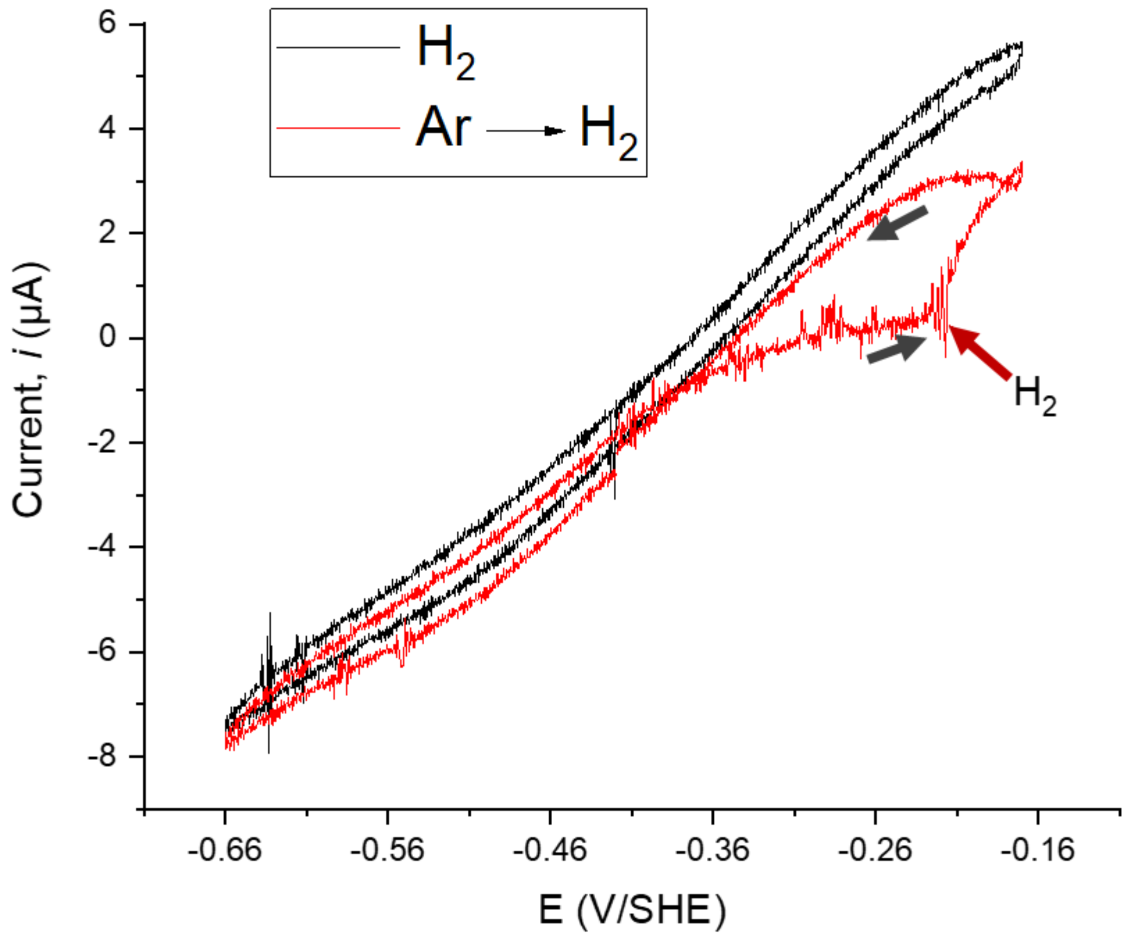

Figure S3. Cyclic voltammetry of *Cr*-HydA1 containing cell lysate from *E. coli*. A scan performed under pure H_2 reveals currents attributable to proton reduction and H_2 oxidation (black). The red trace is recorded under an Ar atmosphere and H_2 is introduced on the upward scan (red arrow) at pH 6.0, room temperature. Scan direction is indicated with black arrows. As seen in the red trace, no oxidative current is observed before the gas switch. Other electrochemical parameters are the same as in figure 4.

Figure S4. Chronoamperometry, *Cr*-HydA1 H_2-dependence

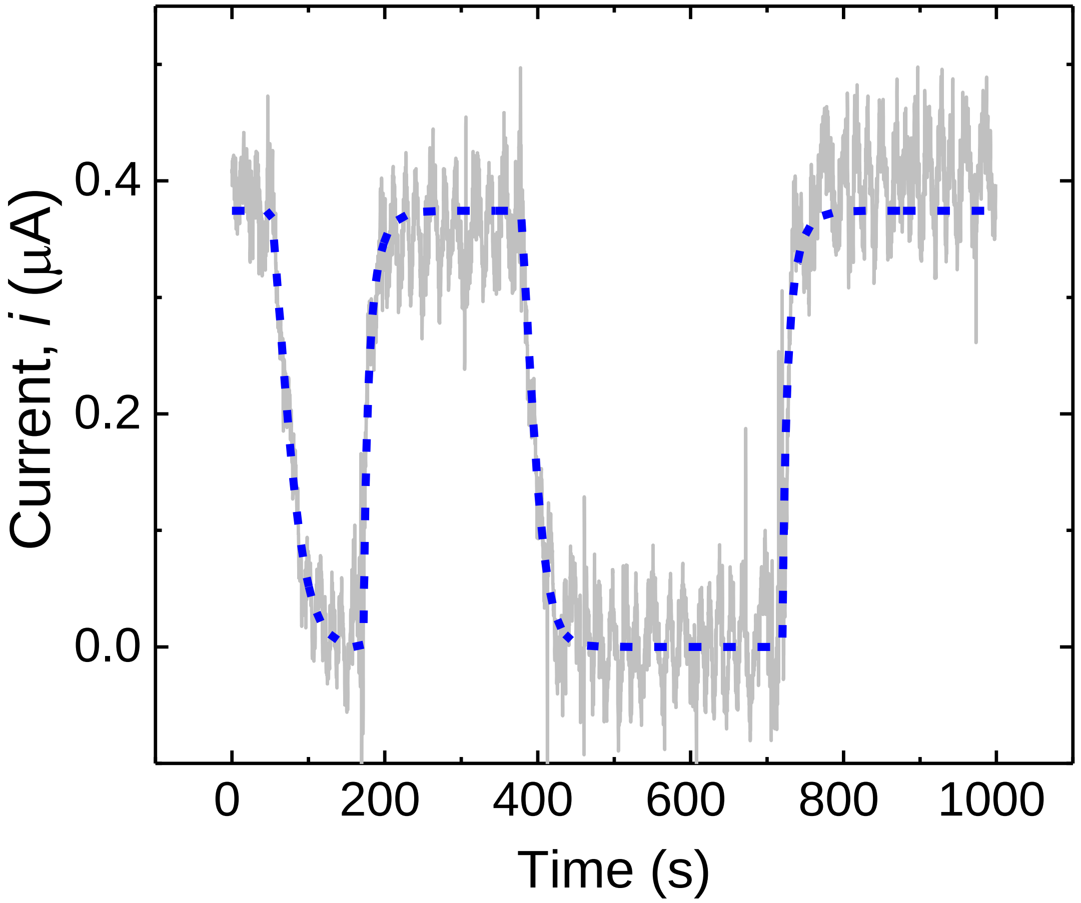

Figure S4. Chronoamperometry of *Cr*-HydA1 containing cell lysate from *E. coli*. H_2 bubbling was swapped with Ar back and forth at t = 80, 180, 390 and 720 s, respectively. The consequent variation of the substrate concentration (from 1 to 0, then to 1 atm, and so on) results in a variation of the catalytic current (grey trace) that can be fitted with the Michaelis-Menten equation with time-dependent adjusted H_2 concentration (blue dashed line).[9] K_M was determined to 0.57 ± 0.15 atm H_2, similarly to previous work using the same method. Experimental parameters: 25°C, pH 7, electrode potential -0.16 V/SHE, electrode rotation rate 3 krpm.

Figure S5. Whole-cell EPR with [2Fe]^{adt} treated *Sm*-HydA and *Tam*-HydA

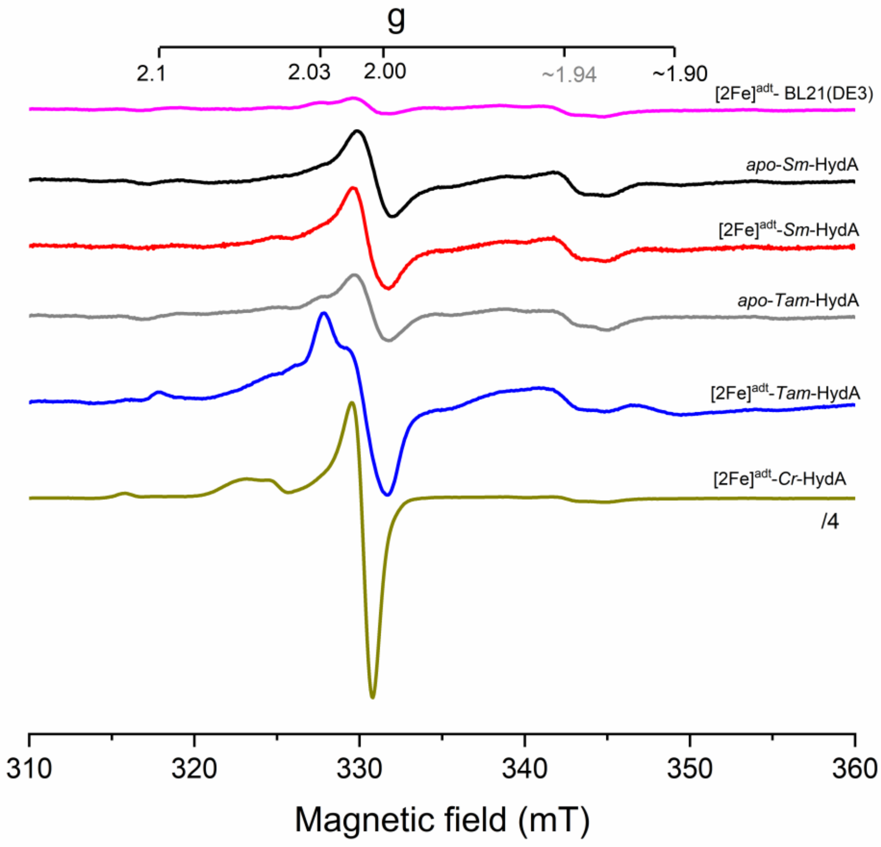

Figure S5. *In vivo* H-cluster assembly using [2Fe]^{adt} was monitored on *E. coli* whole-cells with X-band EPR spectroscopy. Samples were collected from cells incubated in the absence (*apo-Sm*-HydA and *apo-Tam*-HydA) and in the presence of 80 µM [2Fe]^{adt} ([2Fe]^{adt}-*Sm*-HydA, [2Fe]^{adt}-*Tam*-HydA and [2Fe]^{adt}-*Cr*-HydA). No distinct new signals were observed when comparing [2Fe]^{adt}-*Sm*-HydA to *apo-Sm*-HydA. Conversely, a comparison between [2Fe]^{adt}-*Tam*-HydA and *apo-Tam*-HydA reveal the presence of at least two new EPR active species [2Fe]^{adt}-*Tam*-HydA (g-values 2.10; 2.03; 2.00 and ~1.90 indicated with black numbers). The background signals (*apo-Sm*-HydA and *apo-Tam*-HydA) show relatively strong features around g ~ 2.00 and ~ 1.94, complicating a detailed analysis. Still, the peak at g 2.10 strongly support the presence of an H_{ox}-like species,[10] and is most likely connected to the features at 2.03; 2.00. The broad trough observed at g ~1.90 is tentatively attributed to one or more reduced FeS-clusters (sequence analysis suggests that *Tam*-HydA features at least three FeS clusters in addition to the H-cluster). EPR spectra of [2Fe]^{adt} added to BL21(DE3) cells not expressing any [FeFe]-hydrogenase (magenta spectrum) and [2Fe]^{adt}-*Cr*-HydA1 (olive green spectrum) shown for reference. EPR spectra were recorded at 10 K, 1 mW microwave power at a microwave frequency of 9.28 GHz.

Figure S6. Whole-cell EPR with $[2Fe]^{pdt}$ treated *Sm*-HydA and *Tam*-HydA

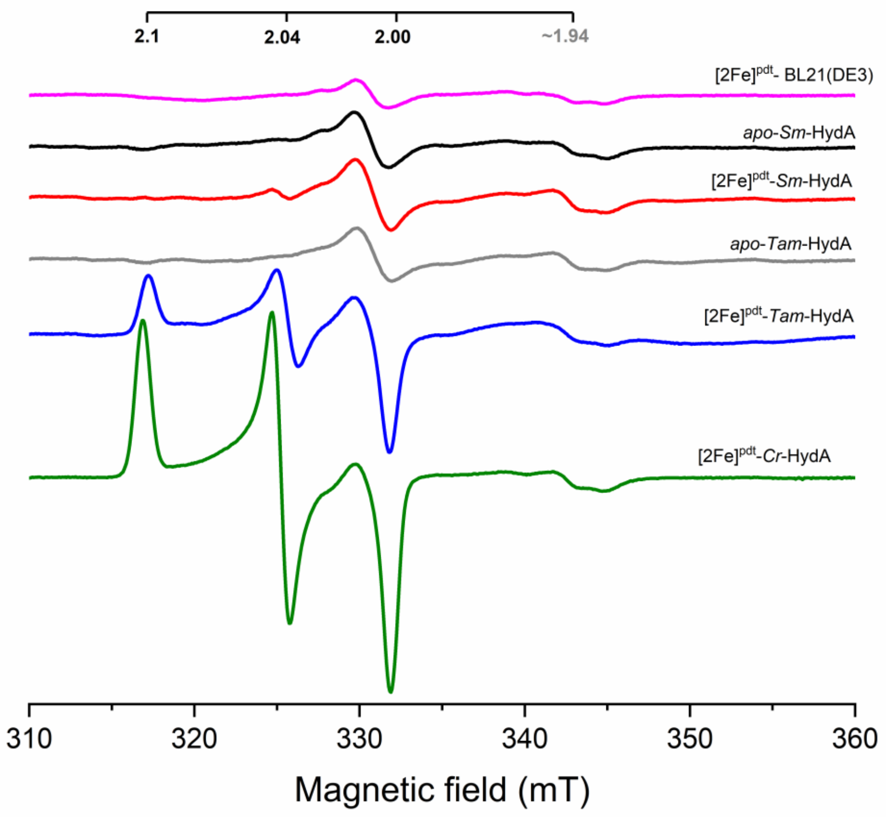

Figure S6. *In vivo* formation of the $[2Fe]^{pdt}$ loaded H-cluster was monitored on *E. coli* whole cells with X-band EPR spectroscopy. Samples were collected from cells incubated in the absence (*apo-Sm*-HydA and *apo-Tam*-HydA) and in the presence of 80 µM $[2Fe]^{pdt}$ ($[2Fe]^{pdt}$-*Sm*-HydA, $[2Fe]^{pdt}$-*Tam*-HydA and $[2Fe]^{pdt}$-*Cr*-HydA). The $[2Fe]^{pdt}$ treated samples show generation of a rhombic signal with g-values = 2.10; 2.04; 2.00 typical for H_{ox} (indicated with black numbers).[10] The intense H_{ox}-like signal of $[2Fe]^{pdt}$-*Tam*-HydA supports the assignment of the g = 2.10; 2.03; 2.00 signal to an H_{ox}-like species also in $[2Fe]^{adt}$-*Tam*-HydA (Figure S4). EPR spectra of $[2Fe]^{pdt}$ added to BL21(DE3) cells not expressing any [FeFe] hydrogenase (magenta spectrum) and $[2Fe]^{pdt}$-*Cr*-HydA1 (green spectrum) shown for reference. EPR spectra were recorded at 10 K, 1 mW microwave power at a microwave frequency of 9.28 GHz.

Figure S7. Whole-cell FTIR spectra

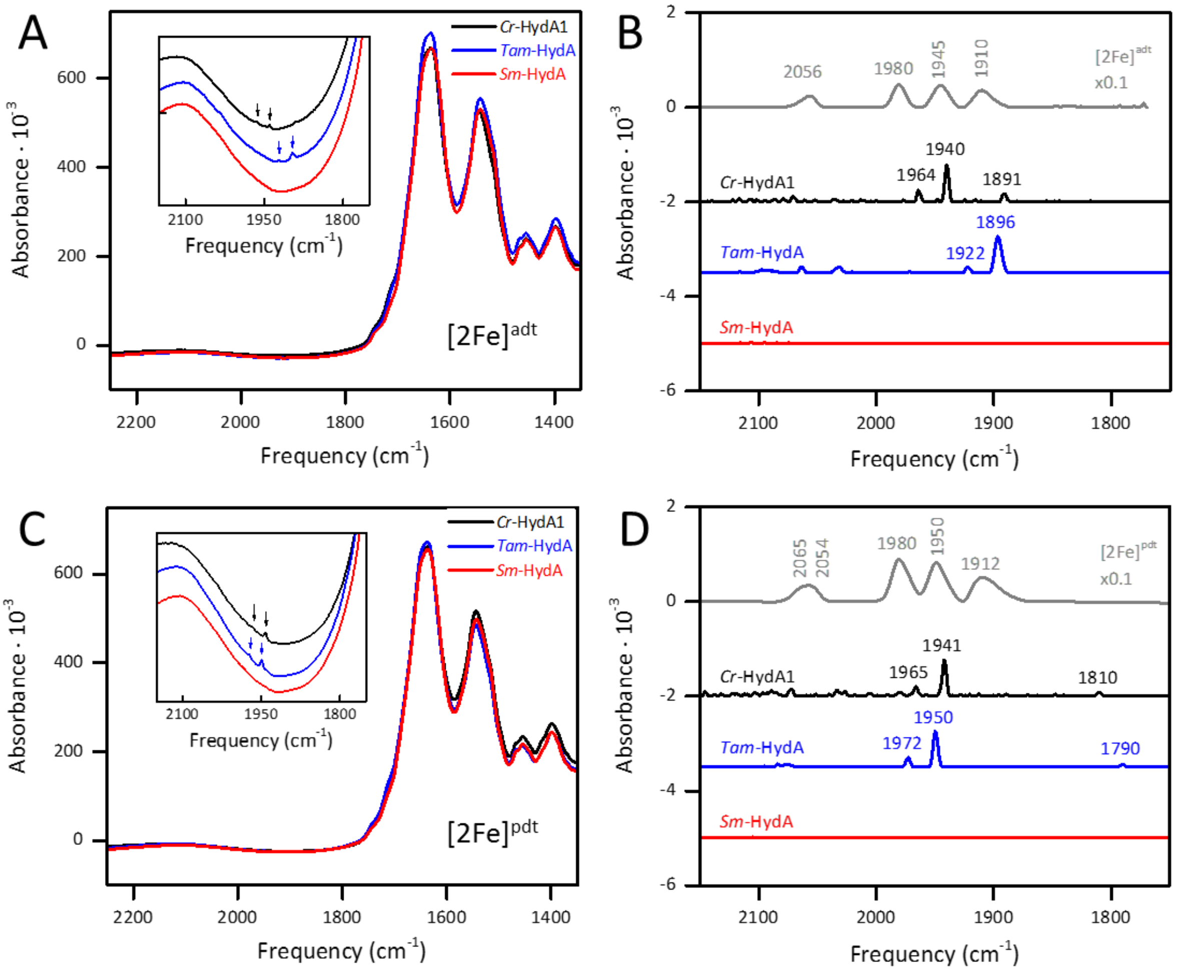

Figure S7. FTIR spectra showing diluted *E. coli* cell suspensions expressing the [FeFe]-hydrogenase from either *C. reinhardtii* (*Cr*-HydA1, black traces), *T. mathranii* (*Tam*-HydA, blue traces), or *S. moorei* (*Sm*-HydA, red traces). *Apo*-protein was activated with either $[2Fe]^{adt}$ (upper row) or $[2Fe]^{pdt}$ (lower row). (**A**) Hydrated cell films (pH 8, under N_2) show comparable levels of protein concentration (amide I at ~1635 cm^{-1} and amide II at ~1540 cm^{-1}). Inset: CO/CN regime of the H-cluster activated with $[2Fe]^{adt}$. The arrows point to bands indicative of functional hydrogenase (note the lack of signal for *Sm*-HydA). (**B**) Baseline-corrected spectra of $[2Fe]^{adt}$ and *E. coli* suspension expressing the [FeFe]-hydrogenase as indicated. The cell films do not show any signs of unincorporated cofactor. The sharp H-cluster bands are easily distinguishable from the broad bands of the [2Fe] subsite mimic. Remarkably, *Tam*-HydA preferentially adopts the one-electron reduced state H_{red} whereas *Cr*-HydA1 rapidly converts into H_{ox} under a N_2 atmosphere. (**C**) Same conditions as in (A), but hydrogenases were activated with $[2Fe]^{pdt}$. Note the lack of signal for *Sm*-HydA. (**D**) Same as in (B), but hydrogenases were activated with $[2Fe]^{pdt}$. Here, *Tam*-HydA and *Cr*-HydA1 both adopt H_{ox}.

Additional information

Sm-HydA amino acid sequence

MSKYQFLDKRVPIADDNISIVQDLSKCKNCTLCRRACAIDAGVFDYYDLTTNGDVPICINCGQCVVSCPFDSLNERSE
LDGVKAAIQDPEKVVVFQTAPAVRVGLGEEFGMPAGTFVQGKMITALRKLGGDYVLDTNFGADMTIMEEASELIE
RVINGNGQLPQYTSCCPAWVKFAETFYPELIPHLSTAKSPIAMQAATEKTYFAKKNNIDPKQIVSVCVTPCTAKKAEI
RRPEMNSSAEYWNEEEMRDSDYCITVRELARWIREAELDFANLEDGKFDPLMGEASGGGIIFANTGGVMESAMR
SAYKFVTKDEVPANLIRFDAIRGFENSREADVQIGDKVLHVAAIHGTGNFRKFYEHMKETGTHYDFIEVMACPGGCI
GGGGMPRHKLPQVKAAKESRIASLYERDKLKPIKISQDNPEIQLLYNEFYGAPLSEKAHHMLHTEGFINRSADLGPN
GACTPETCPTSVANLKKAQQ

Length: 479 amino acids

M_w: 52931 g/mol

Tam-HydA amino acid sequence

MLYFHSVTLDKDRCRGCTNCIKRCPTEAIRVRDGKARIINERCIDCGECIRVCPYHAKLAVTDSLDMMKDFKYKIALP
APSLYGQFRDLTINQILSALLDVGFDEVFEVAYAAEIVSKFTREALAKGNLKKPVISSACPAVVRLVQIRFPSLIDNLLDI
CSPMDTAAILAKKEAIKKTGLKEEEIGVFFISPCAAKVTSVKNPIGIEKSKIDGVFSMKEIYGLIIQKAKTTVVRDLSKAS
MIGVGWANSGGEAFGTFTENSIYVDGIHNVVDVLEEIELGKLNDLDFFEGLACIGGCIGGPLTVENNFVAKNRIRKLT
EKLPKKEEALFDEEEIDFEEVKWKKKIEKSEVMKLDKDISKALEMMKQIDTQYKALPGLDCGSCGSPTCRALAEDIVK
GYATEYDCIFILKDKIKNLSQELNDLAGKIPPVLSDEKE

Length: 435 amino acids

M_w: 48255 g/mol

References

1. H. Li and T. B. Rauchfuss, *J. Am. Chem. Soc.*, 2002, **124**, 726-727.
2. R. Zaffaroni, T. B. Rauchfuss, D. L. Gray, L. De Gioia and G. Zampella, *J. Am. Chem. Soc.*, 2012, **134**, 19260-19269.
3. A. Le Cloirec, S. C. Davies, D. J. Evans, D. L. Hughes, C. J. Pickett, S. P. Best and S. Borg, *Chem. Commun.*, 1999, 2285-2286.
4. M. Schmidt, S. M. Contakes and T. B. Rauchfuss, *J. Am. Chem. Soc.*, 1999, **121**, 9736-9737.
5. E. J. Lyon, I. P. Georgakaki, J. H. Reibenspies and M. Y. Darensbourg, *Angew. Chem. Int. Ed.*, 1999, **38**, 3178-3180.
6. V. Fourmond, *Anal. Chem.*, 2016, **88**, 5050-5052.
7. M. Senger, S. Mebs, J. Duan, O. Shulenina, K. Laun, L. Kertess, F. Wittkamp, U.-P. Apfel, T. Happe, M. Winkler, M. Haumann and S. T. Stripp, *Phys. Chem. Chem. Phys.*, 2018, **20**, 3128-3140.
8. S. Mebs, M. Senger, J. Duan, F. Wittkamp, U.-P. Apfel, T. Happe, M. Winkler, S. T. Stripp and M. Haumann, *J. Am. Chem. Soc.*, 2017, **139**, 12157-12160.
9. V. Fourmond, C. Baffert, K. Sybirna, S. Dementin, A. Abou-Hamdan, I. Meynial-Salles, P. Soucaille, H. Bottin and C. Léger, *Chem. Commun.*, 2013, **49**, 6840-6842.
10. W. Lubitz, H. Ogata, O. Rüdiger and E. Reijerse, *Chem. Rev.*, 2014, **114**, 4081-4148.
